# Effects of Vivo Morpholino Knockdown of Lateral Hypothalamus Orexin/Hypocretin on Renewal of Alcohol Seeking

**DOI:** 10.1371/journal.pone.0110385

**Published:** 2014-10-17

**Authors:** Asheeta A. Prasad, Gavan P. McNally

**Affiliations:** School of Psychology, UNSW Australia, Sydney, New South Wales, Australia; Nathan Kline Institute for Psychiatric Research and New York School of Medicine, United States of America

## Abstract

Two experiments used vivo morpholinos to assess the role of orexin/hypocretin in ABA renewal of extinguished alcohol seeking. Rats were trained to respond for alcoholic beer in a distinctive context, A, and then extinguished in a second distinctive context, B. When rats were tested in the extinction context, ABB, responding was low but when they were tested in the training context, ABA, responding was significantly higher. Microinjection of an orexin/hypocretin antisense vivo morpholino into LH significantly reduced orexin/hypocretin protein expression but had no effect on the ABA renewal of alcohol seeking (Experiment 1). Microinjection of a higher dose of the antisense vivo morpholino into LH also significantly reduced orexin/hypocretin protein expression but this was not selective and yielded significant reduction in melanin -concentrating hormone (MCH) protein expression. This non-selective knockdown did significantly reduce ABA renewal as well as reduce the reacquisition of alcohol seeking. Taken together, these findings show an important role for LH in the ABA renewal of alcohol seeking but that orexin/hypocretin is not necessary for this renewal.

## Introduction

Contexts play a critical role in regulating reinstatement to drug seeking. This role is best shown through the phenomenon of renewal. Crombag and Shaham [1] trained rats to self-administer a combination of heroin and cocaine (speedball) in a distinctive context (context A). Then this responding was extinguished in a second, distinctive context (context B). Finally, rats were tested for reinstatement of drug seeking in the extinction context (ABB) or the training context (ABA). There was evidence for the robust restoration of drug seeking when rats were tested in context A, that is, there was evidence for ABA renewal of drug seeking. There have since been numerous demonstrations of such renewal of drug seeking including for responding based on intravenous heroin [2], cocaine [3] and nicotine [4] as well as for responding for alcohol [5], [6]. This ABA renewal is a robust and reliable form of relapse to drug seeking after extinction training (for reviews see [7], [8]). ABA renewal of extinguished responding is also observed for responding based on natural rewards e.g., [9], [10] and has been observed in numerous other conditioning preparations in rodents e.g., [11]–[13], as well as in human smokers [14] and drinkers [15].

ABA renewal of drug seeking is reasonably well understood at the behavioural level. The finding that drug seeking can be restored with a context change after extinction is consistent with Bouton’s model of extinction learning which states that extinction training supports context-specific learning that masks or inhibits responding in the extinction context but not elsewhere [16], [17]. Hence, this mask is removed and responding is restored when animals are tested outside the extinction context. The neural mechanisms of this renewal are also increasingly well understood. ABA renewal of drug seeking requires a circuitry involving, prefrontal cortex [18]–[20], basolateral amygdala ([18]), paraventricular thalamus [21], ventral tegmental area [2], nucleus accumbens shell [22]–[26], and ventral pallidum [27].

Nonetheless, the roles of other brain regions in this ABA renewal remain less well understood. Of interest to the present experiments was the role of hypothalamic orexin/hypocretin neurons in ABA renewal. The lateral hypothalamus is important for ABA renewal of alcohol seeking that is observed after either extinction [28] or punishment [29] training. Orexin/hypocretin are neuropeptides expressed exclusively in hypothalamus [30], [31] and send projections to a large number of regions forming a broad orexinergic network capable of controlling motivation and arousal [32]. Orexin/hypocretin neurons have been well linked to reward-related behaviors, drug seeking, as well as arousal [33]–[35] and contribute to several forms of reinstatement of drug seeking. For example, (OX_1_R) antagonists attenuate stress [36], [37] and cue-induced [35], [38], [39] reinstatement of alcohol or cocaine seeking. The role of orexin in ABA renewal of drug seeking is less clear. Whereas systemic administrations of an OX_1_R antagonist attenuated ABA renewal of cocaine seeking [40] there have been no reports that an OX_1_R antagonist attenuates ABA renewal to seeking other drugs. There is evidence that ABA renewal of alcohol seeking is associated with significant recruitment (i.e. c-Fos protein expression) of orexin neurons in the lateral hypothalamus [21]. However, these data are correlational and do not show a causal role for orexin/hypocretin in ABA renewal.

The aim of these experiments was to study the causal role of orexin/hypocretin neurons in ABA renewal of alcohol seeking. To do so we used vivo morpholinos directed against orexin/hypocretin mRNA to knockdown orexin protein expression in the lateral hypothalamus [41]. This approach has previously been shown to yield a selective knockdown of orexin protein expression and to prevent the expression of cocaine conditioned place preference [41], [42]. The question of interest here was whether such knockdown of orexin/hypocretin protein expression would attenuate or prevent ABA renewal of alcohol seeking.

## Materials and Methods

### Subjects

7-week-old Male Long–Evans rats (N = 23) (Monash Animal Services, Gippsland, Victoria, Australia) were used for Experiment 1. 7-week-old Sprague Dawley rats (N = 18) (Animal Resource Centre, Perth, Australia) were used in Experiment 2 due to the closure of the animal supplier. Rats were housed in a colony room maintained on 12 h: 12 h light/dark cycle (lights on at 7.00 am) for 1–2 weeks prior to surgery and experimentation. Before surgery, rats were housed in groups of eight (Long-Evans) and groups of 4 (Sprague Dawley). Food and water were available *ad libitum* until 2 days before behavioural training, after which rats were allowed 1 h access to food and water following daily training sessions. Experiments were conducted during the light cycle.

### Ethics Statement

All procedures were approved by the Animal Care and Ethics Committee at The University of New South Wales and conducted in accordance with the NIH *Guide for the Care and Use of Laboratory Animals*. The procedures were designed to minimize the number of animals used.

### Surgery

Rats received intraperitoneal injections (i.p.) with a mixture of 1.3 ml/kg ketamine anaesthetic (Ketapex; Apex Laboratories, Sydney, Australia) at a concentration of 100 mg/ml and 0.3 ml/kg of the muscle relaxant xylazine (Rompun; Bayer, Sydney, Australia) at a concentration of 20 mg/ml. Flat skull coordinates for lateral hypothalamus relative to bregma were −2.5 AP, ±1.8 ML, and −7.5 DV [43]. A 30-gauge needle attached to a 1 µL Hamilton syringe was lowered to the LH. 300 nl of sense or orexin antisense (LLC tools, USA) was injected over 2 min using an infusion pump (KD Scientific) and the needle was left in place for 6 min prior to removal to allow for diffusion. For Experiment 1 only microinfusion procedure was used. For Experiment 2, a combination of microinfusion and cannulation procedures was used whereby rats received bilateral implantation of 11 mm 26 gauge guide cannulae (Plastics One, Roanoke, VA, USA) into AcbSh and also morpholino application to LH. The procedure for morpholino application was the same as Experiment 1. For cannulation, the flat skull coordinates for AcbSh relative to bregma were ±1.35 AP, ±0.75 ML, and −6.5 DV [43]. Guide cannulae were secured to the skull using jeweller’s screw and acrylic cement. Dummy cannulae with dust caps were fitted to guide cannulae to prevent occlusion. Following surgery, rats received intramuscular injection of 0.15 ml of a 300 mg/ml solution of procaine penicillin, 0.1 ml of 100 mg/ml cephazolin sodium, and s.c. injection of 5 mg/kg carprofen. Rats were allowed 5 days to recover from surgery prior to commencement of training, during which time they were monitored daily.

### Vivo morpholino

The antisense sequence for *Rattus norvegicus* prepro-orexin mRNA used was 5′-GTATCTTCGGTGCAGTGGTCCAAAT-3′ and the control was 5′-CCTCTTACCTCATTACAATTTATA-3′ (LLC tools, USA) (Nunez et al, 2009; [41], [44]. The preproorexin precursor is cleaved to orexin A and orexin B. These peptide products exhibit differential affinities for the OX_1_R and OX_2_R receptor 2. The antisense sequence used here reduces expression of both to orexin A and orexin B [41]. All morpholinos were obtained as 2 nmol/µl in 0.5 mM phosphate buffer purchased from LLC tools. In Experiment 1, 0.6 nm (in 0.3 µl) was injected and in Experiment 2, 1.2 nm (in 0.6 µl) was injected.

### Apparatus

All self-administration, extinction training, and tests were conducted in eight standard Med Associates (Med Associates, St Albans, VT, USA) operant chambers, each enclosed in a sound and light-attenuating cabinet equipped with a fan that provided constant ventilation and low-level background noise. For all chambers, front (hinged door) and rear walls were constructed of clear Perspex, and end walls were made of stainless steel. Inside each chamber, two nosepoke holes containing a white cue light were symmetrically located on one sidewall of the chamber, 3 cm above a grid floor. A recessed magazine was located behind a 4×4 cm opening in the centre of the same wall between the two nosepokes. Responding on one (active) nosepoke delivered beer reward to the magazine, whereas responding on the other (inactive) nosepoke had no programmed consequences. To serve as distinct contexts, these sets of chambers were divided into 2 sets of 4. Each set differed in their olfactory, tactile and visual properties. Thus in one set of chambers, 1 ml of dilute peppermint essence was infused into the bedding underneath the chamber floor, the floor was covered in a sheet of Perspex, and the house light, located on the end wall opposite the magazine, was illuminated. In the alternate set of chambers, dilute rose essence was infused into the bedding, the stainless steel bars of the floor were uncovered, and the house light was switched off. These chambers served as context A or B in a counterbalanced manner.

Locomotor activity was assessed in Plexiglas chambers (Med Associates, St Albans, VT, USA) 43.2 cm (width)×43.2 cm (length)×30.5 cm (height) for 40 min. Movement was tracked through the use of three 16 beam infrared arrays. Infrared beams were located on both the X and Y-axes for positional tracking. Food intake was assessed in polycarbonate chambers, 30 cm (width)×28.5 cm (height)×55.5 cm (length).

### Experiment 1 Procedure

There were two groups: Sense (n = 7) and Antisense (n = 16). On Days 1 and 2, rats received two 20 min magazine training sessions per day (once daily in each of contexts A and B) in the self-administration chambers. Rats were trained to nose poke to acquire decarbonated beer, Coopers Birrell’s Premium, 0.5% w/v alcohol content. Pure ethanol was added to the beer so that it resembled full strength beer (adjusted to 4% v/v alcohol). During these sessions, rats received 10 non-contingent deliveries of 0.6 ml of 4% alcoholic beer into the magazine cup at time intervals variable around a mean of 1.2 min. From Days 3 to 9, rats received daily 1 h self-administration sessions in context A. During this phase, responses on the active nosepoke triggered a syringe pump, delivering 0.6 ml of 4% alcoholic beer into the magazine cup on a FR1 schedule of reinforcement, and extinguished the white cue light recessed in the nosepoke during a 24 s timeout. Responses on the inactive nosepoke had no programmed consequences. On Days 10–13, rats were given daily 1 h extinction sessions in context B. Procedures for extinction were identical to self-administration, except that syringes were removed from infusion pumps so that responses on the active nosepoke no longer resulted in the delivery of beer.

On Day 14, one day after extinction training, rats underwent surgery for administration of sense or orexin antisense morpholino to the LH. Surgery was done after acquisition and extinction, but prior to test because the knockdown of orexin protein expression by morpholino is temporary and we wanted to ensure knockdown during test. Five days after surgery, on Day 19–20, rats received a reminder 60 min extinction session in context B.

Rats were tested on Days 20 and 21 (6–7 days post *in vivo* morpholino), once in Context A and once in Context B (counterbalanced) for 60 min during which responding on both the active and inactive nose pokes was recorded, but no reinforcer was delivered.

### Experiment 2 Procedure

There were two groups: Sense (n = 9) and Antisense (n = 9). The procedures for acquisition, extinction (Days 1–13), surgery (Day 15), and reminder extinction (Day 19) were the same as Experiment 1. Rats were tested for expression of extinction (ABB) and renewal (ABA) on Days 20 and 21 (counterbalanced) using the same procedures as Experiment 1.

In addition, rats received additional testing to assess the effects of vivo morpholino orexin/hypocretin knockdown on other measures of motivated behaviour. On Day 21, a minimum of 4 hr after test for ABA renewal or ABB extinction, rats were assessed for food intake using a method similar to [45]. Rats were placed individually in testing chambers for 60 min with pre-weighed portions of rat chow. Paper was placed in the testing chamber to allow measurement of food spillage. At the end of the test, food intake (corrected for spillage) was assessed. Rats had previously been habituated to eating in these chambers via provision of chow in 1 hr sessions on Days 12 (after 3^rd^ day of extinction training) and 20 (after renewal or extinction test). Food intake test days reported are Day 13 (day before surgery) and Day 21.

AcbSh infusions of B/M prevent the expression of extinction and increase expression of c-Fos in orexin/hypocretin neurons [46]. To study whether the vivo morpholino prevented these effects we assessed the effects of AcbSh inactivation in our animals. On Days 22 and 23 rats were tested in context B for the expression of extinction following infusion of saline or the GABA_B_ and GABA_A_ agonists, baclofen (1.0 mM) and muscimol hydrobromide (0.1 mM) (B/M) into AcbSh. One test was preceded by infusion of saline into AcbSh whereas the other test was preceded by infusion of B/M. The compounds were infused in a volume of 0.5 µl at a rate of 0.25 µl/min followed by 2 min for diffusion. The order of testing (Saline versus B/M) was counterbalanced.

On Day 24 rats were assessed for locomotor activity. Rats were placed in locomotor chambers for 40 min.

On Day 24, after completion of locomotor assessment, rats were returned to the self-administration chambers for assessment of reacquisition of alcohol seeking. Rats were returned to context A and tested for self-administration for 1 hr. During this test, responses on the active nosepoke triggered a syringe pump, delivering 0.6 ml of 4% v/v alcoholic beer into the magazine cup on a FR1 schedule of reinforcement, and extinguished the white cue light recessed in the nosepoke during a 24 s timeout. Responses on the inactive nosepoke had no programmed consequences. This was the same procedure used during self-administration training.

### Immunohistochemistry

We used immunohistochemistry to determine orexin and MCH protein levels. We did not quantify mRNA levels of orexin and MCH (but see [41], [42]) because not all mRNA translate into protein forms and so changes in mRNA are not always informative about actual changes in levels of protein. Hence, we assessed protein expression because ultimately this is the most informative about actual orexin and MCH function. At the conclusion of the experiments (corresponding to 7 days post-surgery in Experiment 1 and 9 days post-surgery in Experiment 2), rats were deeply anesthetized with sodium pentobarbital (100 mg/kg, i.p.) and perfused transcardially with 150 ml of 0.9% saline, containing heparin (5000 i.u/ml), followed by 400 ml of 4% paraformaldehyde in 0.1 M phosphate buffer (PB), pH 7.4. Brains were postfixed for 1 h in the same fixative and placed in 20% sucrose solution overnight. Brains were frozen and sliced to 40 µm coronal sections.

Four serially adjacent sets of LH sections were obtained from each brain and stored in 0.1% sodium azide in 0.1 M PBS, pH 7.2. For quantification of orexin immunoreactivity (-IR) and MCH-IR, two separate series of hypothalamic sections were processed using peroxidase immunohistochemistry as the immunoreactivity does not fade over time, allowing clearer quantification. Immunoreactivity for orexin and MCH had to be conducted on adjacent LH sections as they both have cytoplasmic expression. Sections were washed in 0.1 M PB, followed by 50% ethanol, 50% ethanol with 3% hydrogen peroxidase, then 5% normal horse serum (NHS) in PB (30 min each). Sections were then incubated in rabbit antiserum against orexins (1∶20,000; Phoenix Pharmaceuticals) or rabbit antiserum against MCH (1: 1000; Phoenix Pharmaceuticals) in a PB solution containing 2% NHS and 0.2% Triton X-10 (48 h at 4°C). The sections were then washed and incubated in biotinylated donkey anti-rabbit (1∶1000; Jackson ImmunoResearch Laboratories, 24 h at 4°C). Finally, the sections were incubated in avidin-biotinylated horseradish peroxidase complex (Vector Elite kit: 6 µl/ml avidin and 6 µl/ml biotin; Vector Laboratories, 2 h at room temperature), washed in PB, and then incubated (15 min) in a diaminobenzidine solution (DAB) containing 0.1% 3,3-diaminobenzidine, 0.8% D-glucose and 0.016% ammonium chloride. Immunoreactivity was catalysed by the addition of 0.2 µl/ml glucose oxidase (24 mg/ml, 307 U/mg, Sigma-Aldrich). Brain sections were then washed in PB. Sections were mounted onto gelatin-coated slides, dehydrated, cleared in histolene, and coverslipped with Entellan.

All orexin-IR and MCH-IR were counted within the lateral hypothalamus. The structural landmarks from the lateral edge of the fornix to the optic tract were considered as the LH [43], [47]. The total number of orexin-IR and MCH-IR were counted across the entire medial-lateral and dorsal-ventral span of the LH.

### Data analyses

For alcohol seeking data, the number of active and inactive responses during each stage of the experiments were analyzed using ANOVA. To assess orexin knockdown, immunohistochemistry data involving the total counts of labelled neurons per rat were analyzed using ANOVA. For food intake data, the mean amount of food intake in grams was analyzed using ANOVA. Locomotor data was also analyzed using ANOVA. In each analysis, the per comparison error rate (α) was controlled at 0.05.

## Results

### Experiment 1

In this experiment we used a two group design whereby rats received application of a sense or antisense morpholino against orexin/hypocretin mRNA into the lateral hypothalamus to knockdown orexin/hypocretin protein expression. Rats were first trained to self-administer alcoholic beer in a distinctive context, A, prior to extinction in a second context, B. After extinction, and prior to test, rats received application of the vivo morpholinos and then after a recovery period they were tested. Rats were tested once for responding in the extinction (ABB) context and once for responding in the training (ABA) context.

### Histology

**Figure 1:**
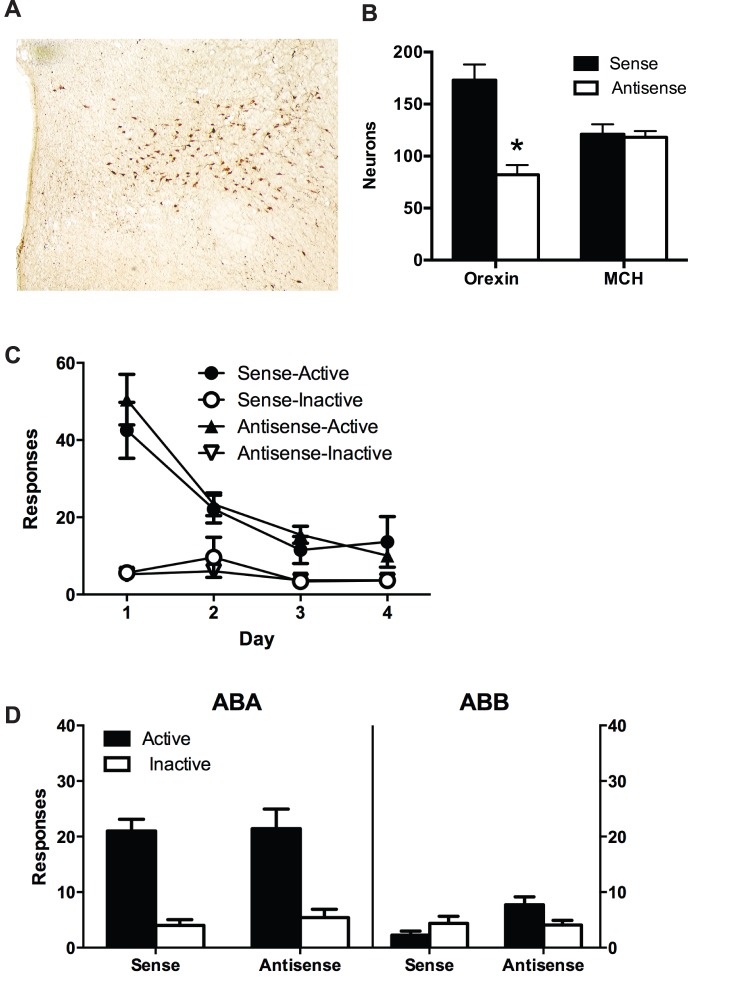
Experiment 1. ***A***, Representative immunostaining for orexin/hypocretin neurons in hypothalamus. ***B***, Quantification of orexin-IR and MCH-IR from separate series of hypothalamic sections processed using peroxidase immunohistochemistry. Mean and SEM numbers of orexin-IR and MCH-IR neurons in LH; ***C***, Mean and SEM active and inactive nosepokes across extinction training; ***D***, Mean and SEM number of active and inactive nosepokes on test for renewal (ABA) and extinction (ABB); *p<.05.

We used immunohistochemistry for orexin/hypocretin and MCH to assess the extent and specificity of morpholino knockdown of orexin/hypocretin protein expression. Figure 1A shows representative photomicrographs depicting orexin/hypocretin-IR in hypothalamus. Figure 1B shows the mean and SEM number of orexin/hypocretin-IR or MCH-IR neurons counted bilaterally in the lateral hypothalamus. The total amount of orexin-IR and MCH-IR was counted across the entire medial-lateral and dorsal-ventral span of the LH. The same structural landmarks for the LH (the region between fornix and the optic tract) were used. There was evidence for the specific knockdown of orexin/hypocretin protein expression by the microinjections of the antisense *vivo* morpholino. There was a significant reduction in the number of orexin/hypocretin-IR neurons in LH for the antisense group compared to the sense group, (*F*_(1, 21)_ = 25.77, p<0.05); but, there was no significant difference between these groups in the number of MCH-IR neurons (*F*_(1, 21)_<1, p>0.05).

### Behavior

We trained rats to respond (nosepoke) for alcoholic beer in a distinctive context (A) then extinguished them in a second context (B) prior to testing in the extinction (ABB) and training contexts (ABA). All rats acquired high levels of responding during training. On the last day of acquisition training, the mean ± SEM numbers of active and inactive nosepokes for the sense group was 111.71±17.33 and 2.28±0.56, respectively. The mean ± SEM numbers of active and inactive nosepokes for the anti-sense group was 117.18±19.73 and 4.12±1.15, respectively. All rats made significantly more active than inactive nosepokes (*F*_(1, 21)_ = 48.80, *p*<0.05) and there were no differences between groups in responding (main effect and interaction, *F*_(1, 21)_<1, *p*>0.05).

The mean ± SEM numbers of responses during extinction training are shown in Figure 1C. All rats made significantly more active than inactive nosepokes, averaged across days of extinction (*F*_(1, 21)_ = 24.89, *p*<0.05). Overall responding decreased across days of extinction (*F*_(1, 21)_ = 44.07, *p*<0.05). This decrease was greater in the active than the inactive nosepoke (*F*_(1, 21)_ = 37.84, *p*<0.05). However, there were no significant differences between groups in responding during extinction (main effect and interaction: *F*_(1, 21)_<1, *p* values>0.05).

Figure 1D shows the mean ± SEM number of responses during tests in the training context (ABA) and the extinction context (ABB). There was no overall difference in responding between the sense and antisense groups, averaged across context and manipulanda, (*F*_(1, 21)_ = 1.15, *p*>0.05). There was overall significantly more responding in the training context (ABA) compared to the extinction context (ABB), averaged across group and manipulanda (*F*_(1, 21)_ = 27.00, *p*<0.05). There was also overall significantly more responding on the active than inactive nosepoke averaged across context and group, (*F*_(1, 21)_ = 16.01, *p*<0.05). Importantly, there was a context×manipulanda interaction showing that the increase in responding when rats were tested in the training context was greater for the active compared to the inactive nosepoke, (*F*_(1, 21)_ = 30.80, *p*<0.05). This shows the ABA renewal of extinguished alcohol seeking. However, there was no effect of orexin knockdown on this ABA renewal. There were no significant between group×manipulanda, group×context, or group×manipulanda×context interactions (all *F*_(1, 21)_<1, *p*>0.05).

### Experiment 2

There was no evidence from Experiment 1 that a selective knockdown of orexin/hypocretin protein expression via infusions of an antisense vivo morpholino had any effect on the ABA renewal of extinguished alcohol seeking. The aim of Experiment 2 was to determine whether any effect on ABA renewal could be observed if a higher dose of the vivo morpholino were used. In addition, given the well-documented role for lateral hypothalamus, and orexin/hypocretin neurons in particular, in feeding, arousal, and other aspects of drug seeking, we employed additional tests to study the effects of the vivo morpholino manipulation. These were assessments of food intake pre versus post knockdown, effects on expression of extinction after AcbSh inactivation, reacquisition of extinguished alcohol-seeking and locomotor activity.

### Histology

**Figure 2:**
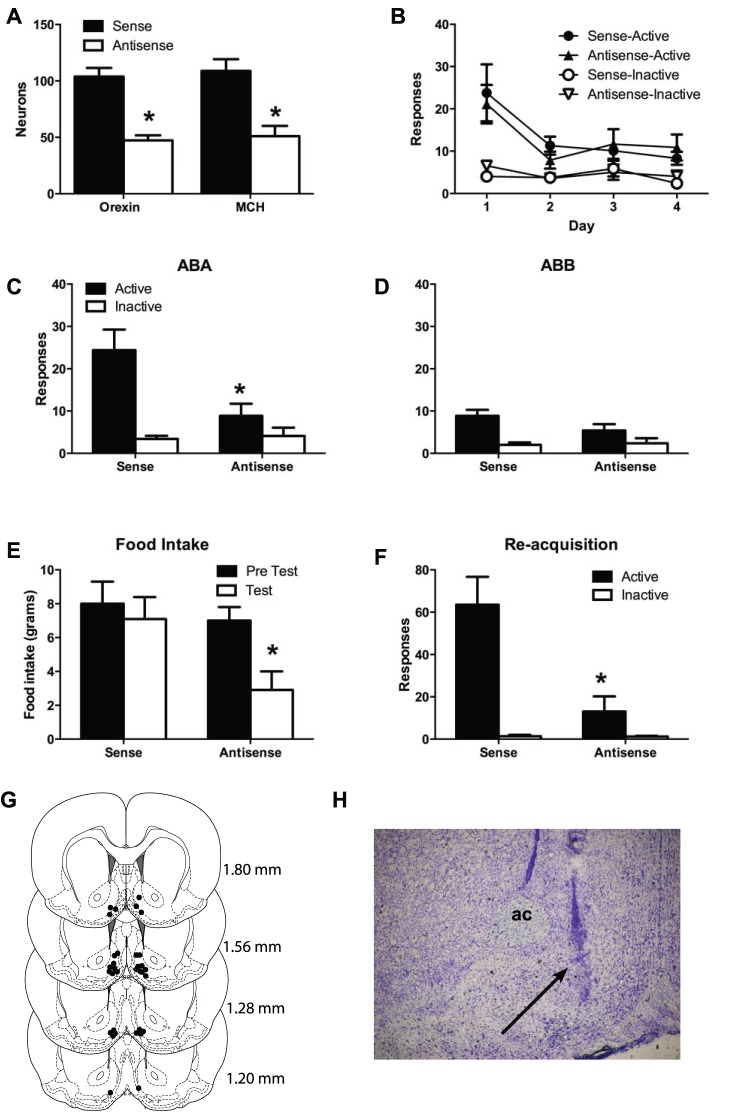
Experiment 2. ***A***, Mean and SEM numbers of orexin-immunoreactive (IR) and MCH-IR neurons in LH from two separate series of hypothalamic sections processed using peroxidase immunohistochemistry.; ***B***, Mean and SEM active and inactive nosepokes across extinction training; ***C***, Mean and SEM number of active and inactive nosepokes on test for renewal (ABA); ***D***, Mean and SEM number of active and inactive nosepokes on test for extinction (ABB); ***E***, Mean and SEM number food intake during pre-test and test; ***F***, Mean and SEM active and inactive nosepokes during re-training of self-administration; p<.05. ***G***, Location of microinjection tips in AcbSh; ***H,*** Photomicrograph of representative microinjection tip in AcbSh.

We used immunohistochemistry for orexin/hypocretin and MCH to assess the extent and specificity of morpholino knockdown of protein expression. Figure 2A shows the mean and SEM number of orexin/hypocretin-IR or MCH-IR neurons counted bilaterally in the lateral hypothalamus. The total number of orexin-IR and MCH-IR were counted across the entire medial-lateral and dorsal-ventral span of the LH. The same structural landmarks for the LH (the region between fornix and the optic tract) were used. There was evidence for the knockdown of orexin/hypocretin protein expression by the microinjections of the antisense *vivo* morpholino, which was approximately of the same magnitude as that observed in Experiment 1. The anatomical extent of this knockdown within the LH was also very similar to that observed in Experiment 1. There was a significant reduction in the number of orexin/hypocretin-IR neurons in LH for the antisense group compared to the sense group, *F*_(1, 16)_ = 21.86, p<0.05. However, in contrast to Experiment 1, this knockdown was not selective. There was also significant reduction in the number of MCH-IR neurons *F*_(1, 16)_ = 7.37, p<0.05, in the animals treated with the orexin/hypocretin antisense morpholino.

### ABA renewal of alcohol seeking

All rats acquired high levels of responding during training. On the last day of acquisition training, the mean ± SEM responses of active and inactive nosepokes for the sense group were 107.44±20.77 and 2.66±0.60, respectively. The mean ± SEM active and inactive nosepokes for the anti-sense group were 113.22±23.95 and 2.44±0.60, respectively. All rats made significantly higher active than inactive nosepokes (*F*_(1, 16)_ = 46.09, *p*<0.05). However, there were no differences between groups in responding (main effect and interaction: *Fs*_(1, 16)_<1, *ps*>0.05).

The mean ± SEM numbers of responses during extinction training are shown in Figure 2B. During extinction training, all rats made significantly more active than inactive nosepokes, averaged across days of extinction (*F*_(1, 16)_ = 53.99, *p*<0.05). Overall responding decreased across days of extinction (*F*_(1, 16)_ = 14.71, *p*<0.05). This decrease was greater in the active than the inactive nosepoke (*F*_(1, 16)_ = 11.29, *p*<0.05). However, there were no significant differences between groups (main effect and interaction: *F*_(1, 16)_ = 0.227, *p* values>0.05) in responding during extinction.

Figure 2C shows the mean ± SEM number of responses during test in the training context (ABA) and Figure 2D shows these responses during test in the extinction context (ABB). There was an overall difference in responding between the sense and antisense groups, averaged across context and manipulanda, (*F*_(1, 16)_ = 10.23, *p*<0.05), so that there was less responding in the antisense compared to the sense group. There was overall significantly more responding in the training context (ABA) compared to the extinction context (ABB), averaged across group and manipulanda (*F*_(1, 16)_ = 18.09, *p*<0.05). There was also overall significantly more responding on the active than inactive nosepoke averaged across context and group, (*F*_(1, 16)_ = 27.93, *p*<0.05). Importantly, there was a context×manipulanda interaction that shows that the increase in responding when rats were tested in the training context was greater for the active compared to the inactive nosepoke, (*F*_(1, 16)_ = 8.81, *p*<0.05). This shows the ABA renewal of extinguished alcohol seeking. There was a significant effect of the antisense morpholino on each of these differences so that there were significant group×manipulanda (*F*_(1, 16)_ = 10.10, *p*<0.05), group×context (*F*_(1, 16)_ = 6.00, *p*<0.05), and group×manipulanda×context interactions (*F*_(1, 16)_ = 5.39, *p*<0.05).

### Expression of extinction after AcbSh inactivation

Others and we have previously reported that AcbSh infusions of B/M prevent the expression of extinction [46], [47] and also that such infusions increase expression of c-Fos in orexin/hypocretin neurons [47]. The question here was whether this reinstatement produced by AcbSh infusions of B/M would be prevented by the antisense morpholino. Rats received two additional tests for the expression of extinction: once after infusion of saline into the AcbSh and once after infusion of B/M. Figure 2G shows the location of microinjection tips in AcbSh and Figure 2 H is a representative photomicrograph of an injection tip in AcbSh. The mean (±SEM) number of nosepokes on saline test was: Antisense = 7.5 (2.7) active, 2.25 (0.8) inactive; Sense = active 3.5 (0.9), inactive 3 (1.9). The mean (±SEM) number of nosepokes on B/M test was: Antisense = 14 (6.0) active, 5.5 (2.3) inactive; Sense- active 8.8 (3.5), inactive 2.9 (0.8). There were significantly more responses on the active than inactive nosepokes, averaged across group and infusion type, (*F*_(1, 16)_ = 9.02, *p*<0.05). However, there was no overall effect of group (*F*_(1, 16)_ = 1.43, *p*>0.05) or infusion type (*F*_(1, 16)_ = 2.66, *p*>0.05). There were also no significant interactions (*Fs*_(1, 16)_<3.04, *ps*>0.05).

### Food intake

Figure 2E shows the mean ± SEM food intake during the pre-knockdown and post-knockdown tests. Averaged across tests there was a main effect for sense versus antisense, (*F*_(1, 16)_ = 4.89, *p*<0.05) averaged across pre versus post-test. There was also a main effect for test, (*F*_(1, 16)_ = 7.68, *p*<0.05) averaged across group. There was also a group×test interaction so that the antisense knockdown significantly reduced food intake during the post-test, (*F*_(1, 16)_ = 11.72 *p*<0.05).

### Reacquisition of alcohol seeking

Figure 2F shows the mean ± SEM responding during the single reacquisition session. In this session rats were returned to the training context and received a single self-administration session identical to the initial self-administration sessions. There was an overall significant difference between groups, (*F*_(1, 16)_ = 11.06, *p*<0.05), showing lower responding among the antisense versus sense group, averaged across active versus inactive nosepoke. There was also an overall effect of manipulanda, (*F*_(1, 16)_ = 24.56, *p*<0.05), so that rats responded significantly more on the active than the inactive nosepoke. Finally, there was a group×manipulanda interaction, (*F*_(1, 16)_ = 11.63, *p*<0.05), so the difference between groups was significantly greater on the active compared to inactive nosepoke.

### Locomotor test

The mean ± SEM distance travelled during the 30 min locomotor test group Antisense mean = 3871.87 cm±668.61 cm, group sense mean = 4820.34±514.42 cm. There was no significant difference between groups in locomotor activity, (*F*_(1, 16)_ = 1.97, *p*>0.05).

## Discussion

These experiments used microinjections of an antisense vivo morpholino to examine the effects of knockdown of orexin/hypocretin protein expression on the ABA renewal of extinguished alcohol seeking. In both experiments there was evidence for the ABA renewal of alcohol seeking. In Experiment 1 there was evidence that the vivo morpholino significantly reduced orexin/hypocretin protein expression in LH. The magnitude of this knockdown is similar to that reported previously using this approach [41], [42]. Nonetheless, despite achieving a 50% knockdown of orexin/hypocretin protein expression there was no impact on the expression of ABA renewal. Both the sense and the antisense groups expressed low levels of responding when tested in the extinction context and significantly higher levels of responding when they were tested in the training context. In Experiment 2, with a higher dose of the antisense vivo morpholino, there was again a knockdown of orexin/hypocretin protein expression. Interestingly, despite using a higher dose, the magnitude of this knockdown (approximately 50%) was the same as that achieved with the low dose vivo morpholino in Experiment 1. However in contrast to Experiment 1, this knockdown was not selective for orexin/hypocretin. The antisense vivo morpholino yielded significant reductions in MCH expression as well, and the magnitude of this MCH knockdown was similar to that of orexin/hypocretin. Under these conditions there was evidence for an attenuation of ABA renewal of alcohol seeking that was selective for responding on the active nosepoke. Moreover, there was evidence for a broader deficit in LH function that included a reduction in food intake as well as a disruption in the reacquisition of alcohol seeking.

Taken together, these findings support three conclusions. First, these results show that the LH is important for ABA renewal of alcohol seeking and show, for the first time, a role in the reacquisition of alcohol seeking. Experiment 2 showed clearly that ABA renewal was significantly reduced in the antisense group. These results are consistent with previous findings that ABA renewal can be attenuated by manipulations that reduce activity of LH neurons [28], [29]. Experiment 2 also showed that the reacquisition of alcohol seeking after extinction training was significantly reduced in the antisense group compared to the sense group. Thus, the LH serves a role in both the renewal and reacquisition of alcohol seeking after extinction training. These effects of the antisense vivo morpholino on ABA renewal and reacquisition do not appear easily attributable to differences in locomotor activity because the sense and antisense groups did not differ in the locomotor assessment.

Nonetheless, and most importantly, these contributions of LH to ABA renewal can be largely independent of orexin/hypocretin. This second conclusion is supported by the findings here that a selective knockdown of LH orexin/hypocretin protein expression had no effect on ABA renewal whereas a knockdown of LH orexin/hypocretin protein expression that also yielded a significant reduction in MCH protein expression did attenuate ABA renewal. This dissociation suggests that although LH is necessary for ABA renewal of alcohol seeking, LH orexin/hypocretin neurons are not. Given the literature reviewed in the introduction, it is not immediately clear why orexin would not be critical for ABA renewal of alcohol seeking. One possibility is that the self-administration procedures and reinforcer used here did not promote sufficient levels of dependence. Past research has shown that OX_1_R antagonists tend to have larger effects on reinstatement of seeking drug rewards than they do on responding for and reinstatement of non-drug rewards [35]. A disadvantage of the OX_1_R antagonist frequently used in the literature, SB-334867, is that it has off-target effects at the adenosine_A2A_ and adenosine_A3_ receptors, the 5-HT_2C_ and 5-HT_2B_ receptors, as well as the monoamine and norepinephrine transporters [48]. However, we have previously shown that LH orexin/hypocretin neurons are recruited during ABA renewal of alcohol [21] but not sucrose [10] seeking. Thus, although LH orexin/hypocretin neurons are recruited during ABA renewal of alcohol seeking, they may not be necessary for this renewal.

Two caveats bear on this conclusion. It is possible that the magnitude of the knockdown produced by the antisense morpholino (approximately 50%) was simply insufficient and that the remaining orexin/hypocretin protein expression was sufficient to support ABA renewal. If this caveat is correct then the results reported here suggest that vivo morpholinos are not useful tools to assess orexin/hypocretin contributions to ABA renewal because they cannot yield both larger and selective reductions in orexin/hypocretin protein levels. Alternatively, it is possible that the co-operative actions of multiple LH neuropeptides are critical for ABA renewal of alcohol seeking and that other neuropeptides may have compensated for the selective reductions in orexin/hypocretin protein in Experiment 1 but were unable to do so in Experiment 2. MCH is an obvious possibility. MCH is critical for cue-induced reinstatement of alcohol seeking [49] as well as of seeking other rewards [50]. MCH protein levels were unaltered in Experiment 1, when renewal was intact, but were significantly reduced in Experiment 2 when renewal was reduced.

The third conclusion is that although the vivo morpholino can yield substantial and selective reductions in orexin/hypocretin protein expression, they can also cause large reductions in other proteins [41]. Here we used MCH as a control because MCH is expressed in a largely non-overlapping group of LH neurons compared to orexin/hypocretin. Our rationale was that any selective effect of the antisense vivo morpholino should be restricted to orexin/hypocretin expression and should not be observed for MCH expression. This was the case for the low dose of the antisense morpholino but it was not the case for the higher dose. This non-selective effect could be due to neurotoxicity but it need not be [41]. For example, electrophysiological studies show that orexins/hypocretins depolarize and increase spiking in MCH neurons, demonstrating that orexin/hypocretin can stimulate MCH neurons [51]. It is possible that the absence or significant reductions of this input to MCH neurons causes a commensurate reduction in MCH expression.

Finally, it is worth commenting on the effects of AcbSh inactivation in Experiment 2. In past experiments, AcbSh inactivation has caused the reinstatement of extinguished cocaine [46] and alcohol [47] seeking. There was no such effect observed here. The reason for this discrepancy is unclear but it is most likely linked to the fact that rats had previously been tested for ABA renewal and had shown robust renewal on that previous test. Prior restoration (e.g., reinstatement, renewal, spontaneous recovery) of extinguished responding reduces, and can abolish, subsequent renewal and response restoration [52]–[55]. Thus, the experimental procedure of testing first for ABA renewal and then for reinstatement by AcbSh inactivation could have weakened or prevented detection of any effect of AcbSh inactivation.

In conclusion, we studied the effects of vivo morpholino knockdown of orexin/hypocretin expression on the ABA renewal of extinguished alcohol seeking. There was no evidence here that selective knockdown of orexin/hypocretin significantly affected ABA renewal. This suggests that, in contrast to cocaine seeking, orexin/hypocretin neurons are not critical for renewal of alcohol seeking. There was a pronounced effect of the antisense vivo morpholino on several measures of drug seeking and appetitive motivation at concentrations that led to non-selective knockdown of both orexin/hypocretin and MCH protein expression. Regardless of the reason, this non-selective effect underscores the need for caution when using vivo morpholinos to manipulate orexin/hypocretin protein levels.
